# An Online Dual-Task Cognitive and Motor Exercise Program for Individuals With Parkinson Disease (PD3 Move Program): Acceptability Study

**DOI:** 10.2196/40325

**Published:** 2022-12-22

**Authors:** Josefa Domingos, John Dean, Júlio Belo Fernandes, Catarina Godinho

**Affiliations:** 1 Department of Neurology Radboud University Medical Center Donders Institute for Brain, Cognition and Behaviour Nijmegen Netherlands; 2 Triad Health AI Aurora, CO United States; 3 Grupo de Patologia Médica Nutrição e Exercício Clínico (PaMNEC) Centro de Investigação Interdisciplinar Egas Moniz Almada Portugal

**Keywords:** Parkinson disease, dual-task training, exercise, digital intervention, online intervention, physical activity, physical therapy, elder, older adult, geriatric, neurodegenerative, adherence, acceptability, community based, group program, online program, physiotherapy, cognitive training online exercise, Parkinson's, neuromuscular, task training, physiotherapist, motor, movement, cognitive, cognition, vocal, voice, speech

## Abstract

**Background:**

Dual-task training is an emerging field used for people with Parkinson disease (PD) to improve their physical and cognitive well-being, but the patients’ acceptability, safety, and adherence to such training in online settings are unknown.

**Objective:**

This study aims to evaluate the acceptability of a dual-task cognitive and motor online training program for people with PD as a group online community program.

**Methods:**

People with PD were invited to participate in an online program (PD3 Move) consisting of physical and vocal exercises in response to different cognitive challenges displayed as dynamic backgrounds on Zoom. The program ran twice per week for 16 weeks. Patient acceptability was assessed at 4 months by monitoring attendance rates and feedback from an exit questionnaire emailed to all participants assessing satisfaction, perceived benefit, safety, and willingness to continue and recommend to others.

**Results:**

The online program was delivered to 15 participants (n=9, 60%, females) with a diagnosis of PD, a mean age of 69.4 (SD 9.3) years, and Hoehn and Yahr (H&Y) stages I-IV. The attendance rate was high, with participants coming to more than 13 (81%) of the sessions. Participants were very satisfied (n=8, 53%) or satisfied (n=7, 47%) with the program. Participants reported that what they most liked were the new cognitive physical challenges. The 3 main facilitators to participating were perceiving the benefits, instructor’s flexibility and engagement, and the social interaction moments with others. The 3 main difficulties were dealing with motor fluctuations (n=3, 20%), difficulties in using technology (n=2, 13%), and difficulty hearing instructions due to hearing loss (n=2, 13%). Patients had favorable perceived benefits of the program, with 14 (93%) considering it very useful for the current management of health and 1 (7%) moderately useful. No adverse events were reported, and all participants said that they were willing to continue the program and recommend it to others.

**Conclusions:**

Our findings suggest that the online cognitive and motor program was well received, safe, and perceived to be of benefit to this group of medically stable people with PD in H&Y stages I-IV. Access to specialized care and enhancement of long-term adherence to regular exercise can be achieved with online community group programs.

## Introduction

Parkinson disease (PD) is considered 1 of the fastest-growing neurological disorders in the world [1]. It causes significant functional disabilities, affecting posture, gait, daily living activities, and cognition [2,3]. Impairments in frontal executive function and attention in PD are common and have been associated with loss of balance and an increased risk of falls [2,4-8].

There is growing evidence suggesting that nonpharmacological interventions, such as exercise/physiotherapy [2,9,10] and cognitive training [11-15], benefit people with PD in both physical and cognitive outcomes. Combining interventions may be a new potential treatment and comes in line with the growing evidence of the feasibility and potential benefits of dual tasking or multitasking in older adults [16,17] and in people with PD [14,18-20].

Initial research in this area of dual-task training concluded that 30 minutes once a week for 3 weeks of multiple-task gait training is feasible in 5 people with PD (Hoehn and Yahr [H&Y] stages I-III, mean age of 61 years with a mean of 8 years with PD) [21], with sustained benefits in multiple-task walking velocity and levels of fatigue and anxiety. More recently, a randomized clinical trial in this area with 121 individuals with early to mid-stage PD showed that dual-task gait improved when compared to a control period without training [19]. The study implemented 2 dual-task training programs, one with consecutive training and the other with concurrent (ie, integrated) dual-task training delivered in the home setting. Importantly, effects transferred to activities in daily life that were not trained and benefits were retained after a 12-week follow-up. This novel training program had excellent compliance from people with PD and did not increase the risk of falls.

Given the recent need for online solutions associated with the coronavirus pandemic and with the advances in technology facilitating access to specialized care [22-25], online exercise programs have emerged as a means for people with PD to stay physically and mentally active [26,27]. Online community-based dual-task exercise programs specifically adapted for people with PD have not been studied yet but may be an excellent tool to facilitate access to the reported benefits of dual-task training.

Here, we aim to assess the acceptability and safety of delivering such dual-task programs in an online group format with people with PD in the early to late stages of PD.

## Methods

### Design

Program acceptability was evaluated with mixed methods quantitative and qualitative assessments [28]. To ensure quality in the research report, we followed the Good Reporting of A Mixed Methods Study (GRAMMS) checklist [29].

### Sampling and Recruitment

The sampling method selection was nonprobabilistic by convenience. Recruitment took place for 3 months from October 2021. All people with PD registered at the Portuguese Parkinson Disease Patient Association were invited to join the program online. People were included if they had (1) a diagnosis of PD (self-reported by patients), (2) H&Y stages I-IV, (3) the ability to connect online via Zoom (Zoom Video Communications, Inc) safely or have a care partner to assist if needed, (4) the ability to communicate with the investigator to understand and comply with the study procedures, and (5) the willingness and ability to provide written informed consent to participate and understand the right to withdraw their consent at any time without prejudice toward future medical care.

Participants were excluded if they had self-reported severe cognitive difficulties and significant active psychiatric problems that would incapacitate them from participating.

A therapist from the patient association clinic carried out the selection process and included participants that met the study criteria via email. The PD3 Move program was provided as an online community exercise program offered by the patient association.

### Program

The program was led by a physiotherapist with expertise in PD and cognitive training, as well as with experience in building and implementing community dual- and multitask programs in PD. The program was delivered in 2 (1-hour) group sessions per week for 16 weeks. The program consisted of combining cognitive exercises projected on a dynamic Zoom background, with participants responding with physical and vocal exercises. The use of different types of Zoom backgrounds to support the cognitive challenge, as well as the use of voice to respond, was expected to be associated with higher levels of engagement, learning benefits, and exercise adherence.

Dual-task training was defined as the capacity to simultaneously perform 2 or more tasks during transfers, ambulation, and other movement-related activities [30]. The performance of these simultaneous 2 attention-demanding tasks had different goals, requiring patients to shift attention between tasks or placing equal amounts of attention on both tasks [31].

The cognitive exercises selected targeted the 4 main cognitive domains (examples in Table 1) particularly affected in PD [32]: attention (ability to apply different cognitive senses), working memory (temporarily storing and managing information), executive function (ability to manage time and attention, switch focus, plan and organize, remember details in sequence), and visual spatial skills (orientation in space, taking in and organizing visual information from the screen).

**Table 1:** Example of 1 exercise per cognitive domain.

Cognitive domain targeted	Exercise instructions	Primary motor goal	Primary cognitive goal
Attention	“Start by stepping in place. Pay attention to random days of the week on the screen and say them out loud. Every time you see Monday, Thursday, and Sunday, lift your arms up.”	Increasing physical capacity, stepping, global amplitude, vocal volume, rhythm, and speed of motor responses	Training sustained and divided attention
Executive function	“Reorganize the activities you see in chronological order as you step in place. 1: I wash my hair; 2: I turn on the shower; 3: I brush my hair.” A video graphic of a 10-second timer is inserted with each new prompt.	Increasing physical capacity and tolerance to dual-task interference of walking/stepping and thinking	Training managing time, planning, and organizing a sequence of activities
Visual spatial	“If you see a dog inside the house, sit down. If the dog is on the left, take a step to the left. If the dog is on the right, take a step to the right. If the dog is in the front, take a step forward.”	Increasing physical capacity, stepping, and tolerance to dual-task interference on quick stepping	Training quick decision-making and working memory
Working memory	“Pay attention to a shopping list of 5 items. Then, with a new background, perform side steps for 30 seconds and then say the names of all [items] on the list.”	Increasing physical capacity and stepping	Retaining information temporarily

Exercises were modified between sessions to maintain motivation and reduce memorization. Physical exercises consisted of an array of frequently recommend movements in PD that directly enhance functional activities of daily living, and relevant to PD, such as sitting and standing, reaching, and stepping or walking in place [2]. All physical exercises focused on high-amplitude, multidirectional movements, increasing in complexity and speed gradually and enough to foster motor learning and motivation but not so quickly as to cause frustration.

Based on the clinical judgment of the instructor and the patient’s performance, exercises were progressively increased through several levels of difficulty via (1) increasing the physical or vocal challenge or (2) manipulation of features on the cognitive exercises (number and type of prompts per time, their intrinsic complexity, or the interval between prompts). Gamification principles were also adapted to some exercises to act as motivational drivers for the participants and enhance social interaction [33]. An example of the organization of an exercise class is given in Table 2.

**Table 2:** An example of the outline of an exercise class.

Phase	Example of a group exercise session
Phase 1: arrival (10 minutes)	Social interaction (eg, greeting friends) Brief discussion on safety issues and how to participate (first timers) Assessment of new health and logistical issues since the last session (second timers)
Phase 2: warm-up (10 minutes)	Group warm-up using whole-body amplitude movements incorporating movements of the neck, shoulders, hands, trunk, hips, and knees, combined with walking in place or in a chair, in a rhythmic routine using appropriate music Voice warm-up exercises with loud ahh, glides, and humming exercises
Phase 3: exercise 1 (learning 5 minutes and training 10 minutes)	Learning movements to respond to a cognitive exercise that will be projected in the background behind the instructor (ie, stand up and sit again every time you see a 5 on the screen; 5 minutes) Training exercise (10 minutes) Progress to more speed-based movement while maintaining amplitude and then to more cognitive demands (ie, lift arms if you see a 3 and count how many fives appear; 5 minutes)
Phase 4: exercise 2 (learning 5 minutes and training 10 minutes)	Learning movements to respond to a cognitive exercise that will be projected (ie, identify the direction of the arrow and take a step in that direction; 5 minutes) Training (10 minutes) Progress to more speed-based movement while maintaining amplitude and then to more cognitive demands (ie, if the arrow is red, do the opposite move; 5 minutes)
Phase 5: fire down (5 minutes)	Active, slow-amplitude movements with music, stretching, and breathing exercises
Phase 6: session assessment (5 minutes)	Brief group discussion to gather participant feedback on each exercise to guide future sessions

### Data Collection

A pretest was first applied to a group of 5 individuals with a diagnosis of PD. Researchers questioned these individuals to understand their perceptions about the online questionnaires. The instrument was considered sufficiently clear, objective, and comprehensive and did not present questions that could be ambiguous or equivocal. This procedure allowed researchers to determine that the survey was suitable for this study.

Before attending the program, all participants completed a questionnaire collecting general information about demographics, clinical problems, past medical conditions, current exercise habits, and perceived facilitators/barriers to exercise. Importantly, when patients reported falls in the assessment questionnaire, they were asked to take safety precautions, namely have a care partner present, always exercise in the sitting position, and signal to the therapist to monitor ongoing risk.

A postassessment anonymous online questionnaire was sent to participants immediately after the program. This questionnaire assessed patients' overall satisfaction, preferences, and barriers and facilitators to participating and any type of problem or adverse events that occurred during the program.

The overall satisfaction with the online program was assessed using a 5-point Likert scale (1=very satisfied, 2=satisfied, 3=neither satisfied nor unsatisfied, 4=unsatisfied, and 5=very unsatisfied). Patients were also asked whether they would recommend the program to a friend (1=yes, 2=no, and 3=maybe) and how likely they are to return to a similar program (1=very likely, 2=likely, 3=neither likely nor unlikely, 4=unlikely, and 5=very unlikely).

To assess the program design, patients were asked which part of the session they preferred: 1=warm-up with simple amplitude-based movements; 2=physical exercises on their own (eg, stepping, raising arms, sit stand); 3=cognitive exercises with movements (answering the cognitive challenges with movement and voice); 4=social interaction moments before, during, and after the session; 5=explanation of the teacher to Parkinson-related questions or reasons for the specific exercise; 6=final relaxation with soft music and breathing exercises; 7=preferred all the parts; and 8=did not prefer any parts.

The instructor assessed patient presence and feedback at the end of each session and prepared a monthly report regarding difficulties or learning points from the application of the program. In addition, the therapist made follow-up phone calls, when needed, to assess people’s feedback, concerns, or any questions. To ensure that the data collected by the instructor remained pertinent to the aims of the study, an interview guide was developed, comprising a set of open-ended questions focused on the difficulties in and facilitators to participating in PD3 Move. Examples of questions used in the guide are:

- Are there any factors regarding technology or other issues limiting your participation in the program?
- Do you have someone to help you during the classes?
- Do you feel the exercises are being delivered at a good speed for you?
- Do you take resting periods, when needed?
- What do you think would assist or facilitate you participation in the exercises?

### Data Analysis

Descriptive statistics was performed using the IBM Statistic Package for the Social Sciences software (SPSS Statistics for Windows, version 27.0). Textual data from open-ended questions and the instructor’s notes were analyzed using the QDA Miner Lite database. The Braun, Clarke, Hayfield, and Terry (2019) process of content analysis was applied. This method of analysis focuses on identifying recurring common themes, ideas, and patterns of meaning in data. This method comprises 4 stages: preanalysis, encoding, categorization, and interpretation of data.

### Ethical Considerations

Researchers followed the principles of the Declaration of Helsinki. The study protocol was approved by the Egas Moniz Research Ethics Board (ID: 948, date: March 25, 2021). Prior to starting the program, all participants received information regarding the study procedures and provided their written informed consent.

The online survey was set up so that participants were free to decide not to answer any question, change or review their responses, or voluntarily quit at any time. To comply with the ethical principles of anonymity and confidentiality, all data collected were free of any personally identifying information, including any form of electronic identifiers.

The archive of essential documents was carried out in a locked file, ensuring their prompt availability, upon request, to competent authorities. All digital data were coded and stored on a password-protected computer. All data will remain locked in a file cabinet at Egas Moniz University for 5 years. After this retention period, all data will be destroyed.

No individual data will be available.

## Results

### Participants

In total, 15 individuals with a diagnosis of PD participated in the program in 16 group sessions of 1 hour each performed twice a week. Participants were mainly female (n=9, 60%) with a mean age of 69.4 (SD 9.3) years and H&Y stages I-IV. The participants’ demographics and clinical characteristics can be found in Table 3.

**Table 3:** Participants’ general and clinical characteristics (N=15).

Characteristics		Value
**Age (years)**		
	Mean (SD)	69.4 (9.3)
	Median	71.0
	Minimum	48
	Maximum	80
**Time since diagnosis (years)**		
	Mean (SD)	10.7 (7.1)
	Median	10.0
	Minimum	3
	Maximum	31
**Sex, n (%)**		
	Female	9 (60)
	Male	6 (40)
**Main problems** **, n (%)**		
	General fatigue	12 (80)
	Urinary problems and constipation	10 (67)
	Difficulties in daily activities	9 (60)
	Difficulties in walking	9 (60)
	Difficulties in balance and falls	7 (47)
	Problems with sleep	7 (47)
	Pain	7 (47)
	Anxiety or apathy or depression	6 (40)
	Difficulties in memory, thinking, and attention	6 (40)
	Tremor	5 (33)
	Difficulties in talking	4 (27)
**Fall history (past 3 months)** **, n (%)**		
	No falls	6 (40)
	1 fall	4 (27)
	2 falls	2 (13)
	Frequent falls	3 (20)

Regarding exercise habits, of the 15 participants, 8 (53%) were doing some form of exercise besides the online program. All participants considered that it is important to exercise regularly, having identified several factors that influence them to exercise, namely the benefits of exercise for their health (n=15, 100%), feeling exacerbation of their symptoms when they do not exercise regularly (n=9, 60%), having fun when exercising (n=6, 40%), and feeling guilty when not exercising (n=2, 13%).

### Program Attendance

The attendance rate was high, with participants coming to more than 13 (81%) of the sessions. People did not attend some sessions, but none totally dropped out of the program. Reasons for not attending reported by participants varied but included medical appointments, health-related issues, and family events. Adherence with the exercises throughout the sessions was also perceived as high by the instructor, with participants continuously engaging in the exercises (assessed via general vocal responses to the exercises).

### Safety

In the exit questionnaire, all participants reported having no major problems during the sessions. Several participants (n=8, 53%) had supervision from family or care partners during the sessions. Based on the instructor’s notes, all participants completed the program, with no report of severe problems during the sessions and some reporting only some fatigue.

### Participants’ Satisfaction and Perceived Benefits

After the program, participants provided favorable feedback, with 8 (53%) feeling very satisfied and 7 (47%) feeling satisfied with the program. In addition, all participants reported their willingness to attend future online classes and said they would recommend it to another person with PD. Patients had favorable perceived benefits of the program (n=15, 100%) for the current management of exercise/health habits, with 14 (93%) considering it very useful and 1 (7%) moderately useful.

### Feedback Regarding the Program Format and Delivery

The frequency of 2 sessions per week was considered ideal by 8 (53%) participants. The remaining participants expressed a preference to participate 3 (n=3, 20%) to 4 (n=4, 27%) times a week in this type of sessions. Regarding the type of physical and cognitive exercises, only 2 (13%) of the participants were familiar with this type of exercises performed. For the remaining 13 (87%) participants, the combination was something new.

Considering all activities carried out, 9 (60%) participants reported enjoying all these activities in general. All participants expressed a preference for 2 components: (1) performing cognitive exercises, responding verbally, and movements and (2) moments of explanation by the health professional regarding Parkinson-related issues or about the reason for the specific exercise. The less preferred components were (1) performing physical movements (eg, walking in place, sitting/rising, taking steps, raising arms; n=1, 7%) and (2) relaxing at the end with soft music (n=1, 7%).

The social moments that allowed the exchange of experiences and ideas was considered useful by 14 (93%) participants, with 1 (7%) participant expressing neutrality in relation to this component of the program.

### Instructor’s Notes Regarding Program Delivery

Instructor notes highlighted that some time had to be allocated to the logistics of the program (ie, helping with technology difficulties, phone calls for follow-up, registering attendance), and this was critical for program success. However, the time requirements necessary decreased as the participants became more agile with the technology and program. Additionally, the instructor reported that the most stressful aspect was the need for constant modification of the exercises during the sessions. This included adjustments to the type of physical activities, the length of the exercise, the use of verbal feedback, the time for learning in a group setting, and resting periods suitable for all. The instructor noted that alerts for the risk of falling and abnormal postural behaviors were needed but occurred mainly at the outset of the sessions and were gradually less needed. Of the 15 participants, 1 (7%) demonstrated increased dyskinesia when dual-tasking, but this was nondisabling. In addition, 3 (20%) participants reported feeling some mental fatigue in the first sessions and additional resting periods were included, alongside less cognitively demanding activities introduced to reduce this discomfort.

### Perceived Difficulties

Of the 15 participants, 10 (67%) expressed having difficulties throughout the program occasionally, 3 (20%) reported experiencing difficulties frequently, and only 2 (13%) were totally comfortable with the program, without feel difficulties. The difficulties most frequently mentioned by the participants were dealing with fluctuations in their health status (n=3, 20%), difficulties in mastering and understanding the technologies necessary to participate (n=2, 13%), difficulties with not being able to listen to instructions due to hearing loss (n=2, 13%), initially feeling confusion and disturbance due to everyone talking at the same time (n=1, 7%), lack of personal motivation (n=2, 13%), and frustration for not being able to perform the exercises (n=1, 7%).

### Perceived Facilitators

The factors most frequently identified by the participants as facilitators to participating in the online exercise program were (1) the dynamics, experience, and professionalism of the health professional who performed the sessions (n=10, 67%); (2) perceiving the benefit of participating and feeling that it improves their well-being (n=10, 67%); (3) feeling motivated to exchange experiences with the group and people with a similar disease (n=10, 67%); (4) mastering the use of technologies (n=5, 33%); (5) suitable timing of the sessions (n=4, 27%); and (6) having the support of family members or caregivers during the sessions (n=4, 27%).

## Discussion

### Principal Findings

There are recognized potential benefits of dual-task exercise for people with PD and calls to include cognitive exercise as a component of comprehensive physiotherapy care [2,20]. However, the patient’s acceptability, preferences, and long-term adherence in online group settings is still unknown. Our findings suggest that the clinician-delivered dual-task motor cognitive program is acceptable, safe, and perceived to be of benefit to this group of medically stable people with PD in H&Y stages I-IV. Online programs will likely remain a key element of future delivery of care for people with PD, and our findings inform that the previously reported feasibility of dual-task training may be replicated in this format and fuel further development of such online community-based programs, resources, and research.

Attendance and satisfaction was high, with people attending 81% of the sessions. This was concordant with previous feasibility studies on online dance therapy programs for patients with PD showing a 100% attendance rate [26].

When compared to in-person programs, online programs can be a way to provide personalized and timely care to more people with PD. This may ultimately impact the change in care models as online therapies can complement in-person care as a means of ongoing and easy access to specialized care.

We believe that online training will be a valid option for disease management in the future as the development of key enabling technologies will allow health professionals to provide training guidance and monitor movement, behavior, and cognitive/motor learning. This state of play will allow real-time assessment of patients' posture and performance, enabling health care professionals to provide timely feedback so that online training contributes more effectively to treating PD [34,35].

Several aspects of this program contributed to the high attendance and satisfaction. First, adherence may be related to highly motivated patients who have continuous encouragement to participate [36]. The phone calls from the therapist when they were absent was appreciated by participants and was a potential motivating factor to come to the next session and may have enhanced adherence [37]. In addition, the familiar relationship that the physiotherapist/instructor had with each of the participants also allowed the therapist to preferentially select the types of exercises that participants would enjoy, while maintaining a strong focus on tasks that would be a match for the physical capabilities of the various participants with PD. This proximity might also explain why all participants indicated that they were willing to continue in the program. Nevertheless, in line with previous studies [19] using dual-task activities, all participants were prepared to continue the same training if it were offered again. Second, the small group size is believed to have enhanced adherence via group social interaction with fellow participants. It allowed for good group dynamics, allowed visual assessment of major safety concerns related to balance limitations, and facilitated follow-up calls. Importantly, even though it is difficult to replicate the social aspects of group in-person classes in online formats, the constant engagement through voice in this program is believed to contribute to greater social interactions. Third, intrinsic motivation, via experiencing and recognizing the actual benefit of the exercises and enjoying participation are important factors for long-term training adherence [38,39]. Fourth, the therapist’s PD-specific and cognitive knowledge was recognized as an important factor to facilitate participation. Additionally, it allowed us to adequately anticipate and act quickly on problems that arose that may have influenced patients’ satisfaction [36,40]. Expertise allowed the physiotherapist to recognize safety issues and to anticipate abnormal postural behaviors that may arise when participants performed additional tasks. Fifth, the program used the participants’ continuous feedback to constantly adapt and develop new exercises.

Importantly, participants were able to bypass common safety concerns and technological difficulties with support from care partners and the instructor. Even though 47% of the participants had reported initially having difficulties with balance and falls and 60% had walking difficulties, no one experienced major problems. This was also in concordance with previous feasibility studies on online programs for PD showing no adverse events [26].

Important insights regarding the effects of combining dual modes of exercise in PD were identified. The physiotherapist/instructor reported that the most stressful problem/factor was the need for constant modification of the exercises during the sessions. Importantly, this constant modification may represent a barrier to replicating programs easily, and further detailed description and research on the exercises delivered should be carried out and shared with others in educational courses [41].

### Limitations

Our study was not without limitations mainly intrinsic to its acceptability nature, small sample size, and single-center design, which imposed restrictions on the generalizability of the findings. Second, although primary data were collected through an anonymous online questionnaire, we should not exclude that the participants' actual reports may diverge from what they revealed due to biases, such as a lack of confidence in guaranteeing anonymity or protecting identity values or beliefs. In addition, we also must consider that if participants perceive it to be socially desirable, they might overstate the frequency of positive items. Third, we included a fairly heterogeneous group of people with PD from different backgrounds and did not speciﬁcally include people with PD with cognitive impairment. Even though it remains to be determined how such people can undergo dual-task training, given the potential benefits of this type of program on cognition due to its cognitively demanding components (memorizing the instructions, quick decision-making to reply, cognitive or dual tasking when dividing attention between physical and cognitive exercises, and dealing with environment with constant changes), these programs may be of particular interest in treating cognitive dysfunction in individuals who already display mild cognitive impairment (to potentially delay or slow down further decline). Future programs should attempt to include individuals with cognitive limitations that reflect the type of frontal lobe deficits that are more impaired in fallers [42]. Importantly, a growing body of evidence suggests that there is an increased risk of falls in the presence of cognitive impairment [5,43,44] as well as in dementia [45,46], and this trend is present in both community-dwelling and institutionalized older populations. Hence, interventions that can potentially improve executive function and cognitive processes, in particular attention, have been recognized as a significant element in the process of treating balance and gait deficits in people with PD [18].

Even so, the use of dual-task training may already be of particular interest in preventing or delaying cognitive dysfunction in people with PD who are not (yet) affected. Additionally, people with PD in early disease stages are more amenable to these types of innovative therapeutic interventions. Any contribution to reduce the obvious pitfalls in such dual motor cognitive interventions will be a valuable adjunct to reduce the evolving needs of people with PD and their care partners. An additional limitation in group settings is the impossibility of effectively monitoring patients during the sessions and registering potential situations of wearing off, sudden offs, patients in off (in other words, patients without the effect of medication), etc. Reaction to the exercises and safety was the primary assessment being conducted during the sessions by the therapist. The type of medication taken by participants is another important issue as the dose and type of pharmacological treatments will highly impact patients’ overall functionality.

### Conclusion

Dual-task training is an emerging field for PD, but access to such specialized care remains limited. Online community-based dual-task exercise programs, such as the PD3 Move program, specifically adapted for people with PD may be an excellent tool to facilitate access to previously reported benefits of dual-task training. It can provide a safe and enjoyable way to reduce physical and cognitive inactivity commonly seen in PD. Yet, the design, type of visual display, type of sessions, and participants included will need further reflection. Sharing the concept of such a program implementation may fuel the development of future research and similar community exercise care services for PD that incorporate the complexity of the cognitive challenges in PD. This ultimately may lead to—at least partly—initial treatment suggestions for those who decide to start such an online program for PD and be useful to guarantee safety and better care to the target population.

## Abbreviations

H&Y: Hoehn and Yahr
PD: Parkinson disease

## Data Availability

The data presented in this study are available on request from the corresponding author.
